# Neoadjuvant volumetric modulated arc radiochemotherapy with a simultaneous integrated boost technique compared to standard chemoradiation for locally advanced rectal cancer

**DOI:** 10.3906/sag-1812-185

**Published:** 2019-10-24

**Authors:** Natalia JANKARASHVILI, Sophio KAKHADZE, Maia TOPESHASHVILI, Lasha TURKIASHVILI, Mariam TCHIABRISHVILI

**Affiliations:** 1 Department of Radiation Oncology, Academician F. Todua Medical Center-Research Institute of Clinical Medicine, Tbilisi Georgia; 2 Department of Radiology, Academician F. Todua Medical Center-Research Institute of Clinical Medicine, Tbilisi Georgia; 3 Department of Surgical Oncology, Academician F. Todua Medical Center-Research Institute of Clinical Medicine, Tbilisi Georgia

**Keywords:** Rectal cancer, chemoradiotherapy, neoadjuvant, boost

## Abstract

**Background/aim:**

The present study aimed to examine whether the combination of neoadjuvant volumetric modulated arc radiotherapy (VMAT) using simultaneous integrated boost (SIB-VMAT) techniques and chemotherapy with capecitabine is associated with better clinical and dosimetric outcomes compared to the standard treatment.

**Materials and methods:**

The study included 59 patients with cT2–T4 rectal cancer. In the standard arm, patients (n = 37) were treated preoperatively with image-guided VMAT plus capecitabine. In the SIB arm, patients (n = 22) were treated with the SIB-VMAT technique plus capecitabine. All patients underwent radical surgical resection after neoadjuvant radiochemotherapy.

**Results:**

In the standard arm, cT0N0 was reached in 12 patients (32.4%), primary tumor clinical downstaging was observed in 22 patients (59.5%), and disease stability was achieved in 3 patients (8.1%). In the SIB arm, cT0N0 was reached in 15 patients (68.2%), primary tumor clinical downstaging was observed in 6 patients (27.3%), and disease stability was achieved in 1 patient (4.5%) (P = 0.028). Complete pathological response was observed in 11 patients (29.7%) in the standard arm and in 13 patients (59.1%) in the SIB arm (P = 0.026). In the SIB arm mild diarrhea appeared in 59.1%, moderate in 40.9%, and severe in 0% of the cases. In the standard arm mild, moderate, and severe diarrhea rates were in 54.1%, 43.2%, and 2.7%, respectively. In the SIB arm mild, moderate, and severe cystitis appeared in 63.6%, 22.7%, and 13.6%, while in the standard group mild cystitis developed in 67.6%, moderate in 24.3%, and severe in 8.1%. Mild, moderate, and severe radiation dermatitis rates were 45.5%, 45.5%, and 9.1% in the SIB group and 40.5%, 48.6%, and 10.8% in the standard group, respectively.

**Conclusion:**

The SIB-VMAT technique is effective and safe for irradiating locally advanced rectal cancer. Its effectiveness is expressed in higher clinical and pathological complete response rates and safety with the same rates of acute toxicity.

## 1. Introduction

Locally advanced rectal cancer (LARC) includes cancers that are extended through the rectal wall and/or have regional lymph nodes involved. Treatment of LARC is associated with great difficulties. Surgery, especially total mesorectal excision (TME), is the most important treatment modality, and in the case of margin-negative resections there is a high chance of cure. However, a multidisciplinary approach including preoperative chemoradiotherapy (CRT) may offer long-term, recurrence-free survival [1].

In the case of LARC, preoperative CRT remains the gold standard of treatment [2]. However, patients treated with abdominoperineal resection may also receive CRT. Many trials have demonstrated the benefits of preoperative CRT with improved compliance, reduced toxicity, and increased local control [3].

There have been improvements in treatment regimens. Radical pelvic radiotherapy (RT) of up to 60 Gy is associated with severe acute and late toxicities such as diarrhea, cystitis, perineal dermatitis, genitourinary dysfunction, and sacral fractures. Lower doses of 40–50 Gy provide good tumor response with acceptable levels of toxicity. For this reason, 45–50 Gy in 25–28 fractions has become established as the standard treatment scheme [4]. Hyperfractionation with acceleration or simultaneous integrated boost (SIB) techniques has also been considered [5,6].

At present for LARC, preoperative CRT followed by surgery is preferred as the best treatment option. The RT field for LARC is recommended to encompass the primary tumor, entire mesorectum, presacral space, and regional lymph nodes [7–10]. There are several clinical trials of preoperative CRT for LARC using SIB-volumetric modulated arc radiotherapy (VMAT), reporting good oncologic outcomes with the same toxicities [11,12]. Based on these clinical data, we applied SIB-VMAT for preoperative CRT in LARC. The aim of using preoperative concurrent chemotherapy and VMAT-RT intensified with SIB dose escalation was to evaluate the resectability and pathological response in early clinical outcomes.

## 2. Materials and methods

### 2.1. Patients’ characteristics

From February 2015 to March 2018, 37 patients with cT2–T4 rectal cancer were treated preoperatively with IGVMAT- 45 Gy in 25 fractions and an additional boost of 5.4 Gy in 3 fractions, plus capecitabine at 825 mg/m2 twice daily. From September 2016 to April 2018, 22 patients with cT2–T4 rectal cancer were treated with the SIB technique, with 46 Gy for the elective volume and 57.5 Gy as a boost to the rectal tumor in 23 fractions, plus capecitabine at 825 mg/m2 twice daily. Patients were included if they did not have any comorbidities, contraindications for radical surgery, or distant metastases, and also if the tumor histopathology was adenocarcinoma.

The median age was 59 years (range: 36 to 84), and of the 59 patients, 33 were male (55.9%) and 26 were female (44.1%). None of them had evidence of distant metastasis (M0). Rectal cancer stage ranged from stage I (T2) to stage III, but most of the patients (72.9%) had stage III rectal cancer (Table 1). The histopathology of all patients (n = 59) was adenocarcinoma. The distribution of tumor differentiation grade (G1, G2, G3) is shown in Table 2.

**Table 1:** Stage distribution between two groups.

			Treatment group		Standard	SIB
			Grade	G1	n	11	9	%	29.7%	40.9%	G2	n	20	12	%	54.1%	54.5%	G3	n	6	1	%	16.2%	4.5%
Total		n			37	22	%	100.0%	100.0%									

**Table 2:** Distribution of tumor differentiation grade within treatment groups.

			Treatment group		Standard	SIB
			Stage	I	n	2	2	%	5.4%	9.1%	II	n	5	7	%	13.5%	31.8%	III	n	30	13	%	81.1%	59.1%
Total		n			37	22	%	100.0%	100.0%									

G1 (Grade 1) - Well differentiated, G2 (Grade 2) - Moderately differentiated, G3 (Grade 3) - Poorly differentiated.

This study was approved by the institutional ethical committee. All participants provided informed consent. The study was conducted according to the Declaration of Helsinki’s “Ethical Principles for Medical Research Involving Human Subjects.”

### 2.2. Simulation technique

All patients were immobilized and treated in the supine position. A supine set-up was associated with more stability and was comfortable for the patient. During the simulation procedure, special marks were painted on the skin, which acted as a target for laser beams. These helped to position the patient’s body for treatment. Simulation was performed with a Siemens Somatom AS CT with slice thickness of 3 mm and was aimed to achieve stable conditions of bladder and rectal filling. This kept the small bowel from migrating into the pelvis and reduced small bowel toxicity.

### 2.3. Contouring

Target volumes and organs at risk (OAR) were delineated on a SomaVision 13.7 (Varian Medical Systems). The gross tumor volume (GTV) was determined by a combination of findings from physical examination, endoscopy, CT, MRI, and/or PET-CT. The primary clinical target volume (CTV) included GTV plus pararectal area. Primary PTV included CTVp plus 5 mm. The CTV node included the internal iliac, presacral, and perirectal nodal groups along with the external iliac nodal region (if lesions extended into gynecologic/genitourinary structures or positive external iliac lymph nodes) and the inguinal nodal region (if lesions extended to the anal verge, perianal skin, or positive inguinal nodes). The PTV node was generated with a 5-mm symmetrical margin around the CTV. The small bowel, bladder, and femoral heads were defined as OAR.

### 2.4. Plan evaluation and radiotherapy procedure

All patients were treated with VMAT technique using the TrueBeam linac system with 6 MV photons and Millennium MLC (120 leaves) (Varian Medical Systems, Palo Alto, CA, USA). Patients were checked daily using CBCT images and were accepted for treatment if the relative variations of the organs between the images were within 3 mm along the three spatial directions.

All VMAT plans were with 2 arcs. The first arc was clockwise with start and stop angles at 181° and 179°, while the second one was anticlockwise with reversed start and stop angles. The collimator angle was set to 10° and 350°. All RapidArc (RA) plans were generated using 6 MV photon beams and modulated with 120 multileaf collimators from a linear accelerator (TrueBeam v.2.5; Varian Medical Systems). Optimizations and dose calculations were performed with the Eclipse treatment planning system (version 13.7). We used the PO 13.7 optimization module (photon optimizer) and analytical anisotropic algorithm to carry out final dose calculation with 2.5-mm grid resolution.

Plan evaluation was performed with dose-volume histogram parameters for target structures as well as for OAR. For target structures, with primary as well as node PTVs, we were investigating V95% (volume of the target, covered by 95% of the prescribed dose) and V100%, D99% (dose to 99% of the treatment volume), and the relative volume exceeding 107% of prescribed dose in PTVp (V107%, i.e. V53.928 Gy). For the bladder we were evaluating V40 Gy (relative volume of the bladder receiving 40 Gy) and Dmax (a point defined as 0.035 mL or less was evaluated as Dmax), V50 Gy and V40 Gy for the small bowel in absolute volume (mL), and V45 Gy for femoral heads.

For assessing dose distribution in the healthy tissue, we reported homogeneity and conformity indexes of the plans. The conformality index for PTVp and PTV node was defined as the volume enclosed by the 95% isodose, divided by the target volume. The homogeneity index of the plans for PTVp and PTV node was calculated as (D2% – D98%) / D50%. For healthy tissue, we also reported the volume of the body minus PTV receiving low doses (V5, V10, and V20 Gy) [13,14].

The plans were optimized to meet the following criteria: bladder volume, receiving 40 Gy less than 50% and no volume should receive 60 Gy; small bowel volume, receiving 50 Gy less than 20 mL and the volume receiving 40 Gy less than 100 mL; femoral head volumes receiving 45 Gy less than 25%.

### 2.5. MRI and surgery

Pelvic MRI was performed after 6 weeks and surgery was performed 8 weeks after completion of preoperative treatment. Tumor size reduction on MRI after 6 weeks was the primary endpoint. The secondary endpoint was postoperative morphologic evaluation. Acute toxicities were evaluated according to the Common Terminology Criteria for Adverse Events (CTCAE), version 3.0, 2003.

### 2.6. Statistical analysis

Statistical analyses were performed using SPSS 16.0. Descriptive statistics were demonstrated as mean ± standard deviations (SD) or median (minimum– maximum) for continuous variables and as a percentage (%) for nominal variables. The continuous variables were compared by independent samples t-test. The chi-square test or Fisher’s exact test was used to compare nominal variables. P < 0.05 was considered statistically significant.

## 3. Results

All patients enrolled in both arms underwent radical surgical resection. Tumor response was evaluated according to the RECIST criteria. R0 resection was achieved in all patients. In the standard arm, cT0N0 was reached in 12 patients (32.4%), primary tumor clinical downstaging was observed in 22 patients (59.5%), and disease stability was achieved in 3 patients (8.1%). In the SIB arm, cT0N0 was reached in 15 patients (68.2%), primary tumor clinical downstaging was observed in 6 patients (27.3%), and disease stability was achieved in 1 patient (4.5%) (P = 0.028). Complete pathological response was observed in 11 patients (29.7%) in the standard arm and in 13 patients (59.1%) in the SIB group (P = 0.026). Toxicity in both arms was the same: gastrointestinal toxicities, skin toxicity, and other genitourinary complications (Table 3).

**Table 3:** Distribution of acute toxicities within treatment groups.

		Treatment group			Standard (n = 37)	SIB (n = 22)	P-value
		Diarrhea	G1	20 (54.1%)	13 (59.1%)	0.706	G2	16 (43.2%)	9 (40.9%)	0.861	G3	1 (2.7%)	0 (0.0%)	0.437
Cystitis	G1		25 (67.6%)	14 (63.6%)	0.758	G2	9 (24.3%)	5 (22.7%)	0.889	G3	3 (8.1%)	3 (13.6%)	0.497
	Dermatitis		G1	15 (40.5%)	10 (45.5%)	0.712	G2	18 (48.6%)	10 (45.5%)	0.812	G3	4 (10.8%)	2 (9.1%)	0.833

Acute toxicities according to Common Terminology Criteria for Adverse Events (CTCAE), version 3.0, 2003. G1 (Grade 1) - Mild AE, G2 (Grade 2) - Moderate AE, G3 (Grade 3) - Severe AE.

For dosimetric parameters, we were investigating V95 (%), V100 (%), D99 (Gy), and V107 (%) for primary as well as for node PTVs. We evaluated mean values for each group and compared the data to each other. In the SIB group, for primary PTV the mean value of V95 (%) was 98.0400, of V100 (%) was 48.8800, of V107 (%) was 0.0164, and of D99 (Gy) was 54.2600. For nodal PTV, in the same group, the mean value of V95 (%) was 99.3560, of V100 (%) was 90.5000, of V107 (%) was 52.1200, and of D99 (Gy) was 44.4400. In the standard group, for primary PTV the mean value of V95 (%) was 99.6000, of V100 (%) was 50.5600, of V107 (%) was 0.0025, and of D99 (Gy) was 48.5000, while for nodal PTV the mean value of V95 (%) was 99.7800, of V100 (%) was 88.3000, of V107 (%) was 43.7400, and of D99 (Gy) was 43.5140.

For the bladder we were evaluating V40 (%) and Dmax (Gy), a point defined as 0.035 mL or less, V50 Gy and V40 Gy for the small bowel in absolute volume (mL), and V45 (%) for the left and right femoral heads. In the SIB group, for the bladder, mean values of V40 (%) and Dmax (Gy) were 47.1760 and 59.1400, respectively. In the same group, for the small bowel, the mean value of V50 (mL) was 19.6000 and V40 (mL) was 94.1600. For the left femoral head, the mean value of V45 (%) was 9.9700, and for the right femoral head V45 (%) was 7.4200. In the standard group, for the bladder, mean values of V40 (%) and Dmax (Gy) were 47.0740 and 51.2660, respectively. In the same group, for the small bowel, the mean value of V50 (mL) was 18.3320 and V40 (mL) was 90.6780, while for the left femoral head the mean value of V45 (%) was 5.0140 and for the right femoral head V45 (%) was 5.1140.

## 4. Discussion

This paper reports the advantages of the SIB-VMAT technique for the treatment of LARC, which is expressed in increased number of pathological complete response and the same levels of acute toxicity. Recent studies have shown that there is a relationship between dose escalation and pathological response after treatment [15,16]. According to some studies, there are high rates of pathological complete response in those patients who receive radiotherapy with high doses, such as 55–60 Gy [17]. Increased number of complete responses will have a significant impact on local recurrence-free and disease-free survival. In many European centers, RT only was used as the neoadjuvant treatment for LARC [18], but two randomized trials have shown better local control rates when adding chemotherapy to neoadjuvant RT [19,20]. Fluorouracil (5- FU) and leucovorin improved complete response and local recurrence rates, but an increase of acute toxicity was also observed [21]. There are some new phase II trials in which new chemotherapy regimens are being tested, such as oral 5-FU [22,23] or oxaliplatin [24] and irinotecan [25,26] in combination with fluorouracil. Not only neoadjuvant chemoradiation but also chemotherapy first following chemoradiotherapy showed good response in case of nonresectable rectal cancer [27]. Other modalities such as intensity-modulated radiotherapy (IMRT) have shown some advantages in cases of rectal cancer. First of all, it is possible to deliver much more concave and uniform dose distributions, which guarantee full conformation to the horseshoe shape of the CTV. IMRT also gives us the opportunity to use a simultaneously integrated boost on GTV to achieve further tumor downstaging without increasing acute toxicities [28]. These and other phase II and III trials are ongoing, but until randomized phase III trials demonstrate improved results, 5-FU-based CRT is the gold standard for locally advanced and recurrent rectal cancer patients [29].

Image-guided treatment delivery using kilo-voltage cone beam computed tomography (kV CBCT), helped to minimize interfraction set-up errors. This gave us the possibility to minimize the set-up margin for targets. Besides high-quality image guidance during treatment delivery, a major advantage of the VMAT technique is the significant reduction in treatment times and sparing the surrounding normal structures. Patient compliance to treatment has been significantly increased. In addition, the time gained can be used to increase patient throughput or to increase image guidance. The SIB-VMAT technique also provides the delivery of higher doses only to the GTV, while the CTV receives standard doses. This leads to better downstaging, increases the number of R0 resections, and provides better dosimetric outcome [30].

The results of this paper show that in rectal tumor irradiation, conformal dose, lower doses to OAR, higher doses to GTV, and faster delivery are achieved by VMAT technique.

Future studies will show if the reduction of normal tissue irradiation is associated with a reduced percentage of late treatment-related toxicity. The regimen used in this study allowed achievement of higher complete and near-complete response rates in the SIB arm despite the advanced stage of the disease, without increased risk of radiation-induced severe acute toxicities. Although longer follow-up is necessary to establish the efficacy of neoadjuvant VMAT radiochemotherapy with SIB, several conclusions are possible from these patients.

In conclusion, we evaluated SIB-VMAT chemoradiotherapy efficacy in the treatment of LARC. The clinical study revealed high efficiency and safety of the described method of treatment. Future studies and followup will show if the high rates of clinical and pathological compete response is associated with a reduced percentage of local recurrence and improvement of disease-free or overall survival rates.

Our study has some limitations. The small number of patients did not allow us to draw a definitive conclusion, and the high rates of clinical and pathological complete response still do not mean that these will have an impact on oncological outcome. The follow-up period is too short to assess the clinical value of the obtained results.

Future neoadjuvant trials also should focus on obtaining tissue from primary tumors and enlarged lymph nodes before and after treatment. With modern molecular biology techniques, evaluation of gene expression and chemotherapeutic resistance markers can be performed. Correlation between resistance markers or other molecular markers and the propensity of cells to metastasize can be identified. Based on these molecular markers and their high accuracy, it will become possible to identify patients for whom neoadjuvant chemotherapy will have the most benefit.
